# A Case of Nivolumab-Induced Severe Mononeuropathy Multiplex and Rhabdomyolysis

**DOI:** 10.1155/2017/1093858

**Published:** 2017-10-22

**Authors:** Katsuya Sakai, Hitoshi Mochizuki, Kosuke Mochida, Kazutaka Shiomi, Masahiro Amano, Masamitsu Nakazato

**Affiliations:** ^1^Division of Neurology, Respirology, Endocrinology and Metabolism, Department of Internal Medicine, University of Miyazaki, Miyazaki, Japan; ^2^Department of Dermatology, University of Miyazaki, Miyazaki, Japan

## Abstract

We report an 81-year-old man with multiple liver metastases after tumorectomy for primary mediastinal malignant melanoma, who experienced limb weakness and sensory disturbance after nivolumab monotherapy. He was diagnosed with nivolumab-induced mononeuropathy multiplex and rhabdomyolysis based on serologic examination, muscle biopsy, magnetic resonance imaging of the limbs, and a nerve conduction study. A course of intravenous methylprednisolone (mPSL) was initiated at 1 g/day for 3 days. After that, oral prednisolone (PSL) was started at 1 mg/kg/day and gradually tapered. Limb muscle strength improved, but when PSL was reduced to 0.3 mg/kg/day, the weakness recurred, and a nerve conduction study showed exacerbation of mononeuropathy multiplex. The patient was again administered intravenous mPSL (0.5 g/day for 3 days) followed by oral PSL at 0.5 mg/kg/day, and his neurological symptoms improved. Nivolumab, an immune checkpoint inhibitor, is used for the treatment of advanced melanoma and other cancers and causes various immune-related adverse events (irAEs). However, neurological irAEs related to nivolumab are rare. Furthermore, there are no reports of simultaneous nerve and muscle impairment. Unexpected irAEs affecting various organs should be recognized and treated appropriately.

## 1. Introduction

Nivolumab, one of the immune checkpoint inhibitors, is a human IgG4 monoclonal antibody to human programmed cell death-1 (PD-1). The drug has significant clinical benefits in the treatment of metastatic melanoma, non–small cell lung cancer, and renal cell carcinoma [1–4]. However, it may cause immune-related adverse events (irAEs) in various organs. Although neurological disturbances due to irAEs are rare [5], our patient suffered from concurrent severe mononeuropathy multiplex and rhabdomyolysis.

## 2. Case Presentation

**Figure 1:**
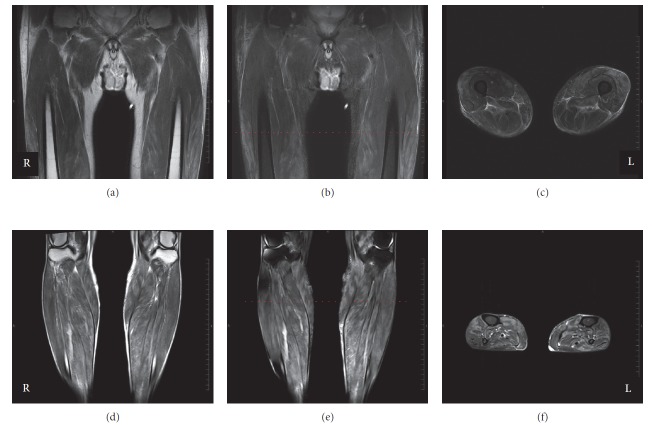
MRI of the lower limbs ((a)–(c) thighs; (d)–(f) legs). T2-weighted ((a) and (d)) and short T1 inversion recovery images (fat suppression method; (b), (c), (e), and (f)). The level of cross-section images ((c) and (f)) is indicated by red dashed lines in the coronal images ((b) and (e)).

An 81-year-old Japanese man with no history of autoimmune disorders and no other significant past medical history underwent tumorectomy for primary anterior mediastinal malignant melanoma, and 4 years later, he was administered nivolumab (3 mg/kg) for multiple liver metastases. On the 8th day after nivolumab administration, he developed symmetric weakness of the proximal muscles of the lower extremities. On the 9th day, he developed further muscle weakness of the left hand and impaired dorsiflexion of the left foot and was admitted to our hospital on the 10th day. Neurological examination showed symmetric proximal muscle weakness of all four limbs, as well as left ulnar nerve and bilateral peroneal nerve palsies. Livedo reticularis was observed on the posterior surface of the bilateral lower legs. Blood tests showed normal levels of urea nitrogen and creatinine but elevated levels of the following: creatine kinase, 27,703 U/L (normal range, 59–248 U/L); aspartate transaminase, 510 U/L (13–30 U/L); alanine transaminase, 157 U/L (10–42 U/L); and lactate dehydrogenase, 811 U/L (124–222 U/L). Thyroid function was within the normal range. Autoantibodies to the following were all negative: acetylcholine receptor, signal recognition particle, gangliosides (GM1, GM2, GM3, GD1a, GD1b, GD3, GT1b, GQ1b, and Gal-C), nuclear antigens, neutrophil cytoplasmic antigens, Jo-1, thyroglobulin, and thyroid peroxidase. Cerebrospinal fluid was negative for malignant cells and showed normal levels of protein (27 mg/dL) and glucose (85 mg/dL). The number of cells was not increased (1/μL). A nerve conduction study showed mononeuropathy multiplex (Table 1); on the 10th day after nivolumab administration, the left ulnar nerve was severely impaired, but the other three nerves of upper limbs were relatively spared. In addition, repetitive stimulation tests (3 and 5 Hz) of the bilateral trapezius muscles showed no abnormalities. T2-weighted magnetic resonance imaging scans with and without fat suppression demonstrated diffuse high signal intensity in the muscles of the lower limbs and partially edematous changes in the subcutaneous tissues (Figure 1()). Skin biopsy of the left lower leg with livedo reticularis demonstrated no specific vasculitis. Pathological examination of the left gastrocnemius revealed various-sized muscle fibers but no necrotic or regenerating fibers. Infiltration of lymphoid cells and neutrophils was not detected. The patient was diagnosed with acute axonal mononeuropathy multiplex and rhabdomyolysis induced by nivolumab. Hydration for the treatment of rhabdomyolysis and intravenous mPSL (1 g/day for 3 days) were started immediately. The intravenous mPSL was followed by oral PSL (1 mg/kg/day), which was gradually tapered. Muscle strength improved slightly. On the 38th day after nivolumab administration, oral PSL was reduced to 0.3 mg/kg/day. On the same day, new-onset weakness was observed in the distal muscles in the distribution of the right median nerve. A nerve conduction study showed a conduction block in the right median nerve (Table 1). This symptom was considered to represent recurrence of the neuropathy without myogenic enzyme elevation, and the patient was therefore administered intravenous methylprednisolone again (0.5 g/day for 3 days), followed by oral prednisolone at 0.5 mg/kg/day. The symptom exacerbation did not progress, and the patient's grip strength recovered. On the 57th day, he was transferred to another hospital for further rehabilitation.

## 3. Discussion

Nivolumab, an anti–PD-1-specific monoclonal antibody, has significant therapeutic effects in the treatment of various cancers, such as metastatic melanoma, non–small cell lung cancer, and renal cell carcinoma [1–4]. However, it is also associated with immune-related adverse events (irAEs) which are attributed to excessive T-cell activation. In our case, nivolumab administration initially resulted in bilaterally symmetric weakness of the proximal muscles accompanied by a prominent elevation of myogenic enzyme levels due to rhabdomyolysis, followed a few days later by mononeuropathy multiplex. Although corticosteroids were somewhat effective, dose reduction resulted in recurrence of mononeuropathy multiplex. Both the muscle and nerve dysfunctions were considered to represent an irAE induced by nivolumab.

Commonly reported irAEs of nivolumab involve the skin, gastrointestinal, hepatic, and endocrine, whereas neurological and muscular irAEs are less common [5, 6]. Chronic inflammatory demyelinating polyradiculoneuropathy, rhabdomyolysis, polymyositis, and myasthenia gravis following nivolumab treatment have been reported [7–9]. Furthermore, since nivolumab acts more specifically on cells expressing the PD-1 ligand than ipilimumab, another immune checkpoint inhibitor that is a monoclonal antibody against cytotoxic T lymphocyte-associated antigen 4 (CTLA-4), irAEs associated with nivolumab seem to be more often localized in organs other than the skin [10]. In our case, however, irAEs induced by nivolumab simultaneously and also sequentially affected multiple organs.

Although corticosteroids are often used to treat irAEs, no standard treatments have been established for rare irAEs such as those affecting muscles and nerves [11]. There are also cases in which plasmapheresis or intravenous immunoglobulin has been effective for neurological or muscular irAEs [12]. In our case, the patient responded to treatment with corticosteroids, but dose reduction resulted in recurrence and worsening of the neurological impairment. Despite the fact that autoantibody tests were negative, the underlying mechanism in this case was considered to be an immune response caused by nivolumab. Steroids may need to be decreased very carefully in such patients.

Nivolumab has remarkable clinical benefits for patients with various cancers and will be used more and more widely in the future. We should recognize the presence of irAEs in various organs when using immune checkpoint inhibitors such as nivolumab.

## Figures and Tables

**Table 1:** Nerve conduction study.

Nerve (right side)			Day of examination	Wrist or ankle latency, ms (amplitude)	Elbow or knee latency, ms (amplitude)	Velocity (m/s)
Median	Right	Motor	10	3.30 (16.7 mV)	6.93 (16.4 mV)	57.9
			38	3.39 (11.6 mV)	8.16 (*2.7 mV*)	45.0
		Sensory	10	2.64 (*2.3 µV*)	—	51.1
			38	2.98 (*3.7 µV*)	—	50.3
	Left	Motor	10	3.51 (3.9 mV)	7.32 (3.5 mV)	52.5
		Sensory	10	2.46 (4.3 µV)	—	64.2
Ulnar	Right	Motor	10	2.67 (7.7 mV)	7.32 (*4.0 mV*)	44.1
			38	2.76 (11.2 mV)	7.32 (10.9 mV)	54.2
		Sensory	10	2.30 (*2.3 µV*)	—	60.9
			38	2.42 (*2.1 µV*)	—	53.7
	Left	Motor	10	2.88 (*0.10 mV*)	8.07 (*0.05 mV*)	*33.7*
		Sensory	10	*n.e.*	—	—
Tibial	Right	Motor	10	4.30 (*0.07 mV*)	*n.e.*	—
Sural	Right	Sensory	10	*n.e.*	—	—

Day of examination, number of days since nivolumab administration; n.e., not evoked. Abnormal data are italicized.
